# African Swine Fever Virus Load in Hematophagous Dipterans Collected in Outbreaks from Romania: Risk Factors and Implications

**DOI:** 10.1155/2023/3548109

**Published:** 2023-02-23

**Authors:** O. M. Balmoș, A. Supeanu, P. Tamba, C. Horváth, L. C. Panait, A. D. Sándor, C. D. Cazan, A. Ungur, M. Motiu, F. A. Manita, B. C. Ancuceanu, F. Bărbuceanu, S. Dhollander, A. D. Mihalca

**Affiliations:** ^1^Department of Parasitology and Parasitic Diseases, Faculty of Veterinary Medicine, University of Agricultural Sciences and Veterinary Medicine of Cluj-Napoca, Calea Mănăștur 3-5, Cluj-Napoca 400372, Romania; ^2^National Sanitary Veterinary and Food Safety Authority, Piața Presei Libere 1, Corp D1, Sector 1, Bucharest 013701, Romania; ^3^Institute for Diagnosis and Animal Health, Strada Dr. Staicovici 63, Sector 5, Bucharest 050557, Romania; ^4^Department of Parasitology and Zoology, University of Veterinary Medicine, Budapest, Hungary; ^5^ELKH-ATE Climate Change New Blood-Sucking Parasites and Vector-Borne Pathogens Research Group, Budapest, Hungary; ^6^Department of Pathology, Faculty of Veterinary Medicine, University of Agricultural Sciences and Veterinary Medicine of Cluj-Napoca, Calea Mănăștur 3-5, Cluj-Napoca 400372, Romania; ^7^European Food Safety Authority, Via Carlo Magno 1A, Parma 43126, Italy

## Abstract

African swine fever (ASF) is a contagious viral disease of swine that causes significant economic damage. The summer peaks and river courses have triggered the hypothesis that vectors may be involved in the transmission of the virus. In temperate climates, insect numbers increase in the late summer. Low temperatures and frosts decrease the number of active insects. Their presence is strongly associated with the nearby wetlands or swamps around the farms. The aim of our study was to evaluate the risk factors associated with the presence of ASFV DNA in hematophagous dipterans and to analyze the relevance of Ct values obtained following RT-PCR analysis of the positive samples in ASF outbreaks in Romania, as an indication for the viral load. The current study included 99 pools of stable flies (*Stomoxys calcitrans*) and 296 pools of biting midges (*Culicoides* spp.), collected in June-September 2020, from 30 outbreaks of ASF in domestic swine from backyard farms (BF), type A farms (TAF), and commercial farms (CF). All extracted DNA was tested for the presence of the ASFV genome using a real-time PCR protocol. Ct values of 39.53 and below were considered as positive (min: 18.19; median: 31.41; max: 39.53). The blood meal source was identified in the hematophagous insects by using a PCR protocol targeting the mitochondrial gene cytochrome c oxidase subunit 1. Data were analyzed using R software v. 4.0.5. In total, 3,158 insects (*S. calcitrans n* = 198 and *Culicoides n* = 2960) were collected in 23 farms of the 30 outbreak farms. Ten species of biting midges were identified. The total number of insect pools showed significant differences according to the month of sampling, with a higher number of pools collected in August and September. Overall, 137 pools out of the 395 examined were positive for the presence of ASFV DNA. There was a higher viral DNA load in farms where pigs were present at the moment of sampling compared to farms where pigs were already culled, in *S. calcitrans* compared to *Culicoides* spp. and in CF and TAF compared to BF.

## 1. Introduction

African swine fever (ASF), caused by the ASF virus (ASFV), is a contagious disease of swine that is associated with a high rate of morbidity, causing hemorrhagic fever-like symptoms and mortality in domestic and wild pigs [1–3]. Natural vertebrate hosts of the virus in Africa are warthogs (*Phacochoerus* spp.) and bushpigs (*Potamochoerus larvatus*), in which infection is unapparent [4]. The virus was introduced from Africa to Europe and South America in the 1950s–1960s. In Spain and Portugal, it remained prevalent for 30 years until eradication. A more recent introduction from Africa to Europe occurred in 2007, when ASFV outbreaks were initially reported in Georgia in the Caucasus region [5]. The first occurrence of this transboundary disease in the vicinity of Romania was reported in 2012 in Ukraine [6]. Since 2014, ASFV has spread across the Baltic States and Poland among wild boars and domestic pigs [7]. Moldova notified the disease as present in September 2016 [8]. With the continuous dispersion, the virus has been reported from Czechia and Romania in 2017 and from Hungary in 2018 [9]. In August 2018, Bulgaria reported the first occurrence of the disease on its territory [8]. Between 2020 and 2022, 16 European Union (EU) and other non-EU European countries have had reported ASF outbreaks in domestic pigs. Among them are Serbia in January 2020 and Germany, where the ASF entered the country in September 2020 [10]. In January 2022, Italy and North Macedonia reported the first occurrence of the disease. ASF has been eradicated in two European countries: Belgium (eradicated in March 2020) and Czechia (eradicated in April 2018) [10].

In Asia, China was the first country affected by the ASFV, with the first cases reported in August 2018. The disease's spread since 2018 has had a great dispersal among the Asian nations, covering almost the whole continent [8]. Since 2005, ASF has been reported in 73 countries throughout the world [10].

ASFV inflicted significant economic damage on Romania's domestic pig sector. In July 2017, the first two outbreaks in domestic pigs were confirmed in Satu Mare County, at the border with Ukraine, in northwestern Romania [9]. The first cases in wild boars were confirmed in Tulcea County, in the southeast of the country, in 2018. The disease has rapidly spread throughout the country, and by 31 October, 2018, 1,073 outbreaks in domestic pigs and 155 in wild boars had been reported. Romania currently stands out as the country with the highest number of confirmed ASF cases in domestic pigs in Europe. In 2021, the country reported 1,660 outbreaks (with ca. 90% of them in domestic pigs), compared to 1,063 in 2020 [11].

The epidemiology of ASF in Romania initially seemed different in comparison to other EU countries, as the majority of outbreaks were reported in domestic pigs and a lower number of cases involved wild boars. Most outbreaks were observed along the Danube River and its major tributaries [9]. Interestingly, ASFV also has been introduced into high-security industrial pig farms. Despite the several hypotheses raised, it is not clear yet which is the most common propagation route for the virus into such facilities [12, 13].

Considering the number of outbreaks, several studies focused on viral transmission in Romanian pig herds. In backyard farms, wild boar population density, growing crops around the farm, domestic outbreaks within 2 km, short distance to wild boar outbreaks, and professional visits were considered high-risk factors [14]. In another study, the presence of water bodies was also found to be an important factor, raising questions regarding waterborne transmission as these waterways nourish backyard properties [15]. It has been shown that immediate quarantine stops infection pathways. Affected farms require effective biosecurity protocols [16].

Soft ticks of the genus *Ornithodoros* have been shown to be competent biological vectors of the ASF virus and are responsible for sporadic spillover of the virus to domestic pigs from the warthog-associated sylvatic cycle in southern and eastern Africa. Here, a cycle involving domestic pigs and similar ticks that live in their shelters has also been described [17]. A similar domestic pig-tick cycle involving another *Ornithodoros* species was responsible for the persistence of infection in the south-western Iberian Peninsula in the last century [18, 19].

The transmission of the virus from the warthog cycle to domestic pigs depends on the *Ornithodoros* ticks because warthogs do not shed virus and are not able to transmit the virus through direct contact. As explained by Jori and Bastos [20], the ticks are not associated with bushpigs, and any natural contact with the bushpig would be incidental. Anderson et al. [21] demonstrated that experimentally infected bushpigs could transmit the virus to ticks, which could in turn transmit it to pigs, but that hardly constitutes “one of the most important routes of infection” since it is almost impossible that it could happen naturally. Spillover from the warthog-tick cycle to domestic pigs occurs occasionally, but a recent study showed that the vast majority of pig outbreaks in Africa including areas where the warthog-tick cycle is present are caused by pigs, people, and fomites and do not involve the ticks at all [22].

The summer peaks and local association with the presence of river courses have triggered the supposition that other vectors maybe involved in the transmission of ASF virus [23, 24]. Flying insects show abundance peaks in the warmest period of the year, and especially in the late summer in temperate climates, insect numbers increase. However, the declining autumn temperatures and especially frosts incrementally decrease the number of active insects [25]. Insect abundance on farms is also strongly influenced by the close neighborhood of wetlands, especially the presence of shallow standing water around the pig farms [26]. The first study, conducted by Mellor et al. in 1987, demonstrated that the stable fly, *Stomoxys calcitrans*, mechanically transmits ASFV after interrupted feeding, suggesting the putative role of other hematophagous dipterans in ASFV transmission. In addition, under experimental condition, Olesen et al. [27] demonstrated that stable flies that feed on viraemic blood can harbor infective doses of ASFV for at least 12 hours. The same authors also demonstrated that under experimental conditions that ingestion of infected stable flies by naïve pigs may lead to infection [28]. Recently, two independent studies demonstrated that ASFV DNA can be detected in *Stomoxys calcitrans* and *Culicoides* spp. collected in ASF outbreaks [29, 30]. However, so far, there is no clear evidence that the virus is actually transmitted mechanically by such vectors under field conditions.

ASFV is routinely detected by using a real-time polymerase chain reaction (real-time PCR) [31–34]. The target DNA is amplified and identified in real time by fluorescence emission during the PCR procedure. After several, sequential amplification cycles, the amount of fluorescence produced by a PCR reaction tube containing the amplified DNA target will exceed a predefined level; this number is known as the cycle threshold (Ct) value [35]. A Ct value may be used to assess a sample's viral load [36].

The present manuscript includes data presented in the project report for EFSA [29], completed with a considerable number of insect pools not analyzed therein while performing statistical analysis and using a different approach for data interpretation (the inclusion of the quantitative Ct values). The aim of this study was to systematically evaluate the risk factors associated with the presence of ASFV DNA in hematophagous dipterans and to analyze the relevance of Ct values of the positive samples in ASF outbreaks in Romania as an indication for the viral load.

## 2. Materials and Methods

### 2.1. Samples: Collection and Pooling

The current study includes arthropod samples collected in June-September 2020 from 30 outbreaks of ASF in domestic swine from backyard farms (BF) (small, subsistence farms with low levels of biosecurity and producing only for personal consumption), type A farms (TAF) (medium-sized farms with some biosecurity procedures and capable of delivering animals to commercial abattoirs), and commercial farms (CF) (high levels of biosecurity and capable of delivering animals to commercial abattoirs). A set of inclusion criteria that prioritized localities (communes) based on a variety of parameters and a scoring system were used for their selection. All the sampling details, protocols, and methodologies are described in Balmoș et al. [29]. Literature recommendations were followed to establish the sampling design in case of all these groups. Each farm was sampled once. Five mini CDC UV light traps per farm were used for collecting *Culicoides* spp. [37]. The traps were placed inside the farms; the collection was conducted during the night. Traps were switched on 1 h before sunset and switched off 1 h after sunrise.

The trapping method we used for *S. calcitrans* was based on sticky traps, commercial traps known as Knight Stick. On each farm, two different locations were chosen. The traps were placed outside, near the animals, at a distance of maximum 15 m and at 1.6 m height. The collection was conducted during the daylight, as stable flies are active during the day and attracted to translucent or blue-coloured objects. All stable flies and biting midges were preserved in 70% ethanol and kept at −20°C prior to morphological identification and testing for ASFV DNA presence and blood meal source. From all the collected arthropods, only stable flies (*Stomoxys calcitrans*) and biting midges (*Culicoides* spp.) were included in the current analysis. The identification of arthropods was based on morphological characteristics, using keys and descriptions [38, 39].

Pools were made prior to DNA extraction. Pooling was conducted based on the insect species, collection date, and farm. Due to size differences among insect groups, stable flies were tested in pools of 2 insects per pool and biting midges in 10 insects per pool, as previously suggested [40, 41]. Pools were only created if the minimum number of insects per pool (2 or 10) was available.

The 395 pools (99 for *S. calcitrans* and 296 for *Culicoides* spp.) included in the current study are detailed in Table 1.

### 2.2. Real-Time PCR

All extracted DNA was tested for the presence of the ASFV genome at the National Reference Laboratory (IDAH-Institute for Diagnosis and Animal Health) using a real-time PCR protocol, according to Standard Operating Procedure (SOP) Identification of the ASFV genome based on the European Union Reference Laboratory for African Swine Fever (ASF EURL) SOPs [33]. DNA extraction was performed at IDAH, individually from each pool, according to the same SOPs.

Pooled samples were ground in MagNa Lyser Green Beads with 1.5 ml cold phosphate buffered saline (PBS1x) supplemented with 0.1% of antibiotic (gentamicin sulphate). Suspensions were clarified by centrifugation at 5,000 g during 5 min, and 200 *μ*l supernatant was used immediately for ASFV genome detection or stored at <−70°C until further use.

A Ct value was obtained for each positive sample. This value indirectly characterizes the viral load, by providing quantitative data for the amount of ASFV DNA. For this study, we considered samples with Ct values of 39.53 and below as positive.

### 2.3. Identification of the Blood Meal Source

Blood meal analysis was conducted in order to evaluate if the vectors feed on pigs or on other hosts and to check if the ASFV DNA positive vectors have fed on pigs. However, our method is not able to distinguish between wild and domestic pigs. Nevertheless, wild boars were not present at the site of vector sampling during our work. The PCR amplification targeted the cytochrome c oxidase subunit 1 (COI) gene region (∼758 bp). The reaction was performed in 25 *μ*l total volume, containing 12.5 *μ*l MyTaq™ Red Mix (Meridian, Teltow, Germany), 6.5 *μ*l of ultrapure water, 1 *μ*l (10 pmol/*μ*L) of each of the two primers M13BCV-FW (5′- TGT AAA ACG ACG GCC AGT HAA YCA YAA RGA YAT YGG -3′) and BCV-RV1 (5′- GCY CAN ACY ATN CCY ATR T -3′) [42], and 4 *μ*l aliquot of the previously isolated DNA. One negative control consisting inultra-pure water was included. The PCR reaction was performed using the T1000™ Thermal Cycler (Bio-Rad, London, UK) with the following conditions: initial denaturation at 95°C for 2 min, followed by 35 cycles of denaturation at 95°C for 1 min, annealing at 45°C for 1 min, and extension at 72°C for 1 min, with a final extension at 72°C for 5 min. The first PCR amplification was followed by a second reaction (nested PCR) performed in 25 *μ*l total volume, containing 12.5 *μ*l Red PCR Mastermix (Rovalab GmBH, Teltow, Germany), 8.5 *μ*l of ultrapure water, 1 *μ*l (10 pmol/*μ*L) of each of the two primers M13 (5′- TGT AAA ACG ACG GCC AGT -3′) and BCV-RV2 (5′- ACY ATN CCY ATR TAN CCR AAN GG -3′) [42], and 2 *μ*l of the initial PCR reaction mix. A second negative control consisting in ultrapure water was included. The second amplification (nested PCR) was performed with the following conditions: initial denaturation at 95°C for 2 min, denaturation at 95°C for 1 min, followed by 15 cycles of a touch down protocol decreasing the annealing temperature from 60°C to 45°C for 1 min (−1°C/cycle), and extension at 72°C for 1 min, followed by another 25 cycles of initial denaturation at 95°C for 2 min, denaturation at 95°C for 1 min, annealing at 45°C for 1, and extension at 72°C for 1 min, with a final extension at 72°C for 5 min.

The amplified products were visualized by the use of electrophoresis on 1.5% agarose gel stained with ECO Safe 20,000× Nucleic Acid Staining Solution (Pacific Image Electronics, New Taipei, Taiwan), and their molecular weight was compared to a molecular marker (O'GeneRuler™ 100 bp DNA Ladder, Thermo Fisher Scientific Inc., Waltham, MA, USA). All positive PCR products were purified using the ISOLATE II PCR and Gel Kit (Bioline Meridian Bioscience, Luckenwalde Germany) and sequenced by the use of an external service (Macrogen Europe, Amsterdam, the Netherlands). All positive sequences were compared with those available in GenBank™ by the use of Basic Local Alignments Tool (BLAST) analyses. All positive sequences were analyzed and edited by the use of Geneious ® 4.85 software [43].

### 2.4. Statistical Analysis and Mapping

Data were analyzed using R software v. 4.0.5. The Shapiro–Wilkinson normality test was used to evaluate the distribution of numerical data. The observed prevalence of infection related to each category and its 95 confidence interval (95% CI; Wald confidence interval) were calculated. Statistical associations between different categorical variables were assessed using Pearson's chi-squared and chi-square goodness of fit tests. The relationship between the month of sampling, farm type, presence/absence of pigs at the moment of sampling, presence/absence of pig DNA in the vector pool, and insect species on one side and Ct values on the other side was evaluated by the Mann–Whitney *U* test and Kruskal–Wallis rank sum test. To test for colinearity and combined effects of multiple predictors, we ran a logistic regression, where farm location was included as a random effect. Ct values were considered both as quantitative variables and categorical data when classified based on the positivity degree (>30 weakly positive, 30–24 positive, and <24 strongly positive). The phi (Φ) coefficient was used to analyze the strength of association between the presence/absence of pigs or the insect genus and the presence of ASFV. Results were considered statistically significant at a *P* value< 0.05. The maps were generated using QGis 3.6.2 software (https://www.qgis.org).

## 3. Results

### 3.1. Collected Arthropods

In total, 3,158 insects (*Stomoxys calcitrans n* = 198 and *Culicoides n* = 2960) were collected in 23 outbreak farms out of the 30 sampled farms (in 7 farms, no *Stomoxys* or *Culicoides* were collected). These resulted in a total number of 395 pools. Based on the morphological characteristics, 10 species of biting midges were identified, divided into 296 pools (Table 2). The stable flies collected were all identified as *Stomoxys calcitrans* (Table 3). The number of insects per pool is shown in “Supplementary file 1_Number of insects per each pool.”

The total number of insect pools showed significant differences according to the month of sampling, with a higher number of pools collected in August and September (*X*^2^ = 262.51, d.f. = 3, and *P* < 0.001). The total number of *Culicoides* pools was significantly higher in August and September, when compared to July (*X*^2^ = 242.92, d.f. = 3, and *P* < 0.001). Moreover, a higher number of *Culicoides obsoletus* (*X*^2^ = 391.09, d.f. = 9, and *P* < 0.01) was observed when compared to the other *Culicoides* spp. The total number of *S. calcitrans* pools was higher in August when compared to the other months of sampling (*X*^2^ = 84.636, d.f. = 3, and *P* < 0.01).

### 3.2. ASFV DNA Presence

**Figure 1:**
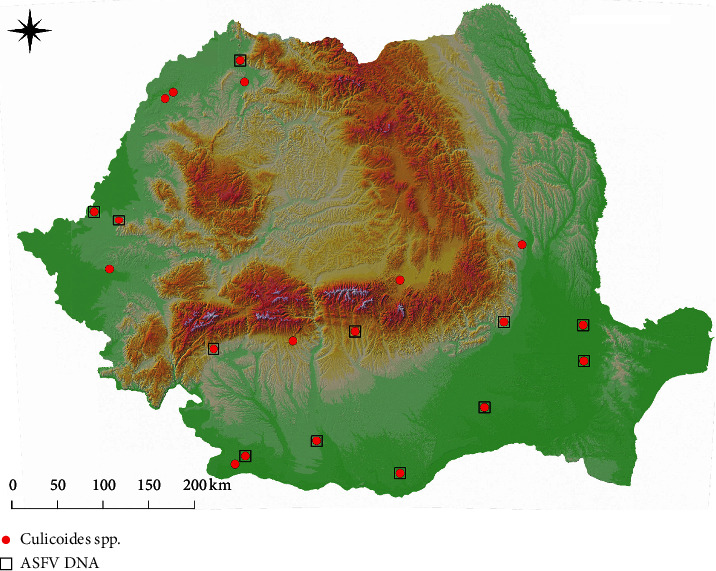
The distribution of sampling sites for *Culicoides* spp. and the positivity to ASF DNA.

**Figure 2:**
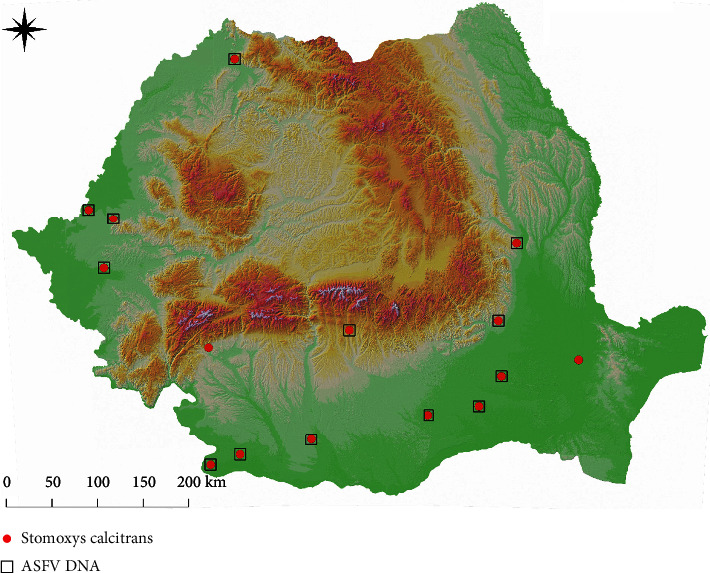
The distribution of sampling sites for *Stomoxys calcitrans* and the positivity to ASF DNA.

Overall, 137 pools out of the 395 pools examined (34.68%, 95% CI: 29.99–39.38) were positive for the presence of ASFV DNA. By the insect group, 79 out of 296 pools of *Culicoides* spp. (26.69%, 95% CI: 21.65–31.73) were positive for the presence of ASFV DNA, with positive samples for each species. Out of 20 locations where *Culicoides* spp. were sampled, ASFV DNA positive pools were found in 12 (60%) (Figure 1). *Stomoxys calcitrans* was collected in 15 farms. ASFV DNA positive pools were detected in 13 of these (86.67%) (Figure 2). Overall, 58.59% (95% CI: 48.88–68.29) (58/99) pools were positive for ASFV DNA.

There was a statistically significant correlation between the overall prevalence of ASFV DNA positive pools according to the month of sampling (*X*^2^ = 41.969, d.f. = 3, and *P* < 0.001), with the highest prevalence in August. A lower rate of infection was observed in case of BF and TAF, when compared to cu CF (*X*^2^ = 106.22, d.f. = 2, and *P* < 0.001). A statistically significant correlation was also identified between positive ASFV DNA pools and the insect group (*Culicoides* spp. vs. *S. calcitrans*) (*X*^2^ = 33.318, d.f. = 1, and *P* < 0.001) with higher rates recorded in *Stomoxys calcitrans* (fair positive correlation; Φ = 0.29).

The prevalence of ASFV DNA positive pools was significantly higher (*X*^2^ = 94.003, d.f. = 1, and *P* < 0.001) in farms where sampling was performed when pigs were still present (60.45%, 95% CI: 53.25–67.66) compared to farms where pigs were already culled by the time of sampling (13.76%, 95% CI: 9.19–18.33). There was a moderate correlation (Φ = −0.49) between the presence of ASFV DNA positive pools and the presence of living pigs. However, only pig absence/presence (*Z* < 0.001) and the month of sampling (*Z* = 0.1998) were retained as significant predictors of ASF DNA positivity, when the combined effect of multiple predictors was tested (logistic regression, farm location used as a random effect).

Out of 395 pools, 51 (12.91%; 95% CI: 9.60–16.22) contained vertebrate DNA, with pig DNA detected in 37 pools (9.37%; 95% CI: 6.49–12.24). Pig DNA was detected only in vectors collected in August from two CF farms where pigs were still present at the time of sampling and one TAF where the pigs were already culled at the time of sampling. Pig DNA was detected in four species of vectors: *C. obsoletus*, *C. punctatus*, *C. pulicaris*, and *Stomoxys calcitrans*. However, no statistically significant differences were noticed between species.

All pools positive for pig DNA were also positive for ASFV DNA. From the pools negative for pig DNA, 19.43% were positive in the case of *Culicoides* and 55.91% for *S. calcitrans*.

### 3.3. ASFV DNA Ct Values

Obtained CT values of the positive samples ranged from 18.19 to 39.53, with a median value of 31.41 and a mean value of 30.14. When Ct values (as quantitative data) were correlated with the presence or absence of pigs, significantly lower Ct values (corresponding to higher viral DNA load) were obtained in farms where pigs were present during sampling (Mann–Whitney *U* test, *W* = 2599.5, and *P* < 0.001).

Moreover, when the categorical data were taken into consideration, the absence of pigs in the farms was statistically associated with a higher number of weak results and no strongly positive results were obtained (Pearson's chi-square test, *X*^2^ = 15.966, d.f. = 2, and *P* < 0.001).

Regarding the insect group (*Culicoides* spp. and *S. calcitrans*), significantly lower Ct values were obtained in *S. calcitrans* than in *Culicoides* spp., meaning a higher viral DNA load in the previous one (Mann–Whitney *U*-test, *W* = 3042, and *P*=0.001).

Strongly positive Ct values (as categorical data) were significantly more common in *S. calcitrans* than in *Culicoides* spp. (Pearson's chi-square test, *X*^2^ = 43.732, d.f. = 2, and *P* < 0.001).

Moreover, significantly lower Ct values were observed in CF and TAF, than in BF (Kruskal–Wallis chi-squared = 14.485, d.f. = 2, and *P* < 0.001).

The Pearson chi-squared test has been used to determine the correlation between the category of Ct values and the type of farm. More weakly positive results were obtained in BF, while in TAF and CF, positive and strongly positive results were predominant. The results are considered statistically significant with a *P* value of 0.03 (*X*^2^ = 11.057, d.f. = 4).

A significantly higher number of strongly positive pools (Ct values below 24) were noticed in June, while weak results (Ct values above 30) were predominant in pools from September (Pearson's chi-square test, *X*^2^ = 25.342, d.f. = 6, and *P* < 0.001).

The Kruskal–Wallis test was performed in order to perform the correlation between Ct values categories and the sampling month of the insects. No statistically significant results were obtained.

Moreover, no significant association was identified between the three categories of Ct values and the presence of pig DNA.

## 4. Discussion

The presence of ASFV DNA in hematophagous insects was demonstrated on several occasions, under experimental [27, 28, 44] or field conditions [29, 30]. Understanding the risk factors associated with the presence of ASFV DNA in hematophagous insects which are demonstrated as mechanical vectors for other diseases (i.e., *Culicoides*, *Stomoxys*) represents the basis for such complex experimental studies.

This was experimentally demonstrated for ASFV by *S. calcitrans* [44]. Biting midges can transmit a variety of arboviruses because of their recurring need for blood meals [45]. *Culicoides* ecology, their presence in pig farms [46, 47], and ability to mechanically transmit pathogens make them potential vectors for ASFV mechanical transmission. *Culicoides* spp. are able to feed on pigs [48]. Although they ingest very small quantities of blood (approx. 1 *μ*l) and get very low viral doses from infected animals, their abundant presence may facilitate mechanical transmission and infection of healthy animals due to frequent bites and injection of sufficient viral doses. Moreover, in the eventuality of infection, their high capacity for passive dispersal may contribute to virus transmission [24]. However, no studies have been conducted to assess if *Culicoides* spp. can transmit ASF.

In a recent study from Lithuania, Turčinavičienė et al. [30] reported the presence of ASF DNA in *Stomoxys calcitrans*, Culicidae, Calliphoridae, and other Muscidae, but no correlation of Ct values was performed, and no risk factors were analyzed. Similar qualitative data are available from Poland, where ASFV DNA was detected in *S. calcitrans *Mazur-Panasiuk et al., unpublished data in [49]. Herm et al. [12] demonstrated the presence of ASFV DNA in flies (*Musca domestica*, *Drosophila* spp.) and mosquitoes collected in ASF outbreaks in Estonia; however, high Ct values were obtained.

In addition, Forth et al. [50] demonstrated the presence of ASFV DNA in two blowfly species larvae under experimental conditions, however without isolating infectious virus.

Other field studies did not identify ASF DNA in hematophagous or other arthropods. Yoon et al. [51] reported no traces of ASFV DNA in a large sample set of arthropods (Diptera: Muscidae, Calliphoridae, Culicidae, Ceratopogonidae, Tipulidae, Scathophagidae, Sarcophagidae, Chironomidae, Psychodidae, Stratiomyidae, Blattaria, Ixodidae, Lepidoptera, Coleoptera, and other unclassified arthropods) collected from ASF outbreaks in South Korea. Herm et al. [52] assessed the detection of ASFV in various blood-feeding arthropods (*Ixodes ricinus*, *Culicoides* spp., Culicidae, and Tabanidae) collected at wild boar baiting sites in Estonia. No positive results were obtained. Qin et al. [53], using the same quantitative approach to identify ASFV DNA, obtained negative results in more than 400 mosquitoes sampled from five ASF outbreaks in China.

Up to date, the involvement of *S. calcitrans* or *Culicoides* spp. in the mechanical transmission of ASFV has never been demonstrated under field conditions. Our study is the first one to evaluate indirectly the viral load (by assessing the Ct values) in stable flies and biting midges collected from ASF outbreaks and to evaluate the risk factors associated with the viral DNA presence and quantity in these insects. Our study demonstrated that there is a higher viral DNA load in (1) farms where pigs were present at the moment of sampling compared to farms were pigs were already culled; (2) *S. calcitrans* compared to *Culicoides* spp.; (3) CF and TAF compared to BF.

Additionally, we demonstrated that the possibility of finding ASFV DNA in hematophagous dipterans collected from ASF outbreaks is higher in (1) commercial farms compared with other types of farms; (2) farms where pigs are still present at the time of sampling; (3) insects collected during August; (4) *Stomoxys* compared with *Culicoides*. The possibility of finding pig DNA in hematophagous dipterans blood meal collected from ASF outbreaks was higher in (1) vectors collected in farms where pigs were present at the moment of sampling; (2) ASFV DNA positive samples; (3) August compared to June, July, or September.

Both *Culicoides* and *Stomoxys* are able to take their blood meal on a variety of host species, which are more available in a BF than in a CF or TAF. Moreover, at least for *Stomoxys*, an individual insect can feed on multiple hosts before being fully engorged. Hypothetically, another element could contribute in contradiction with an interesting hypothesis: ASF-affected pigs could be preferred feeding hosts due to their impaired mobility due to disease.

An accurate understanding of the novel mechanical ASFV vectors is important because it could give a better idea of how this virus can be spread, making it easier to control outbreaks and protect pig farms, among other diseases as well.

In conclusion, our data bring new evidence for the possible role of *S. calcitrans* and *Culicoides* spp. as mechanical vectors for ASFV, highlighting the risk associated with higher viral loads which could improve the approach to the prevention and control, mainly in commercial farms through a better management of biting insects [54].

## Figures and Tables

**Table 1:** Pools of *S. calcitrans* and *Culicoides* spp. used for this study.

Species	Month				Type of farm			Presence of pigs at sampling		Total
	Jun	Jul	Aug	Sep	BF	TAF	CF	Pigs	No pigs	
*Culicoides* (all species)	10	4	142	140	145	47	104	132	164	296
*Stomoxys calcitrans*	12	16	64	7	27	36	36	45	54	99
Total	22	20	206	147	172	83	140	177	218	395

BF: backyard farms; TAF: type “A” farm; CF: commercial farm.

**Table 2:** Number of pools of *Culicoides* per species by the month of collection, type of farm (CF = commercial farm; TAF = type A farm; BF = backyard farm), and presence of pigs at the time of sampling.

Species	Month				Type of farm			Presence of pigs		Total
	Jun	Jul	Aug	Sep	BF	TAF	CF	Pigs	No pigs	
Localities	4	3	9	4	10	5	5	6	14	20
*C. obsoletus*	4	2	76	38	54	8	58	61	59	120
*C. newsteadi*	1	0	11	14	13	8	5	11	15	26
*C. punctatus*	2	0	18	24	4	10	30	31	13	44
*C. nubeculosus*	2	0	24	18	27	14	3	17	27	44
*C. circumscriptus*	2	0	2	8	9	2	1	3	9	12
*C. festivipennis*	0	0	1	6	5	0	2	4	3	7
*C. lupicaris*	0	0	5	29	29	5	0	0	34	34
*C. pulicaris*	0	0	5	1	1	0	5	5	1	6
*C. puncticollis*	0	0	0	1	1	0	0	0	1	1
*C. submaritimus*	0	1	0	1	2	0	0	0	2	2
Total pools	10	4	142	140	145	47	104	132	164	296

BF: backyard farms; TAF: type “A” farm; CF: commercial farm.

**Table 3:** Number of localities and pools where *Stomoxys calcitrans* was sampled.

Parameters	Month				Type of farm			Presence of pigs		Total
	Jun	Jul	Aug	Sep	BF	TAF	CF	Pigs	No pigs	
Localities	2	3	7	3	5	6	4	6	9	15
Pools	12	16	64	7	27	36	36	45	54	99

BF: backyard farms; TAF: type “A” farm; CF: commercial farm.

## Data Availability

All supporting data are available in the current study or can be accessed upon a request from the corresponding author.

## Supplementary Materials

Supplementary Materials Supplementary file 1_Number of insects in each pool.
