# Supplementary material for: African Swine Fever Virus Load in Hematophagous Dipterans Collected in Outbreaks from Romania: Risk Factors and Implications
Source: Transbound Emerg Dis. 2023 Feb 23;2023:3548109. doi: 10.1155/2023/3548109 (PMC12017034; doi:10.1155/2023/3548109)
Supplement: Supplementary Materials — Supplementary file 1_Number of insects in each pool. [file 3548109.f1.docx]

| **Locality code** | **County** | **Vector** | **Number of individuals/pool** | **Ct value** | **grade of positivity** | **Farm type** | **Month** | **Pigs presence** | **Blood source** |
| --- | --- | --- | --- | --- | --- | --- | --- | --- | --- |
| RO-Tac-BR | BR | *Stomoxys calcitrans* | 2 | 0 |  | TAF | august | no |  |
| RO-Tac-BR | BR | *Stomoxys calcitrans* | 2 | 0 |  | TAF | august | no |  |
| RO-Tac-BR | BR | *Stomoxys calcitrans* | 2 | 0 |  | TAF | august | no |  |
| RO-Tac-BR | BR | *Stomoxys calcitrans* | 2 | 0 |  | TAF | august | no |  |
| RO-Tac-BR | BR | *Stomoxys calcitrans* | 2 | 0 |  | TAF | august | no |  |
| RO-Tac-BR | BR | *Stomoxys calcitrans* | 2 | 0 |  | TAF | august | no |  |
| RO-Tac-BR | BR | *Culicoides obsoletus* | 10 | 0 |  | TAF | august | no |  |
| RO-Tac-BR | BR | *Culicoides circumscriptus* | 10 | 0 |  | TAF | august | no |  |
| RO-Tac-BR | BR | *Culicoides lupicaris* | 10 | 0 |  | TAF | august | no |  |
| RO-Tac-BR | BR | *Culicoides lupicaris* | 10 | 0 |  | TAF | august | no |  |
| RO-Tac-BR | BR | *Culicoides lupicaris* | 10 | 0 |  | TAF | august | no |  |
| RO-Tac-BR | BR | *Culicoides lupicaris* | 10 | 0 |  | TAF | august | no |  |
| RO-Tac-BR | BR | *Culicoides lupicaris* | 10 | 0 |  | TAF | august | no |  |
| RO-Tac-BR | BR | *Culicoides nubeculosus* | 10 | 0 |  | TAF | august | no |  |
| RO-Tac-BR | BR | *Culicoides nubeculosus* | 10 | 0 |  | TAF | august | no |  |
| RO-Tac-BR | BR | *Culicoides nubeculosus* | 10 | 0 |  | TAF | august | no |  |
| RO-Tac-BR | BR | *Culicoides nubeculosus* | 10 | 0 |  | TAF | august | no |  |
| RO-Tac-BR | BR | *Culicoides nubeculosus* | 10 | 0 |  | TAF | august | no |  |
| RO-Tac-BR | BR | *Culicoides newsteadi* | 10 | 0 |  | TAF | august | no |  |
| RO-Tac-BR | BR | *Culicoides newsteadi* | 10 | 0 |  | TAF | august | no |  |
| RO-Tac-BR | BR | *Culicoides newsteadi* | 10 | 0 |  | TAF | august | no |  |
| RO-Tac-BR | BR | *Culicoides punctatus* | 10 | 0 |  | TAF | august | no |  |
| RO-Tac-BR | BR | *Culicoides punctatus* | 10 | 0 |  | TAF | august | no |  |
| RO-Tac-BR | BR | *Culicoides punctatus* | 10 | 0 |  | TAF | august | no |  |
| RO-Tac-BR | BR | *Culicoides punctatus* | 10 | 0 |  | TAF | august | no |  |
| RO-Tac-BR | BR | *Culicoides punctatus* | 10 | 38.18 | **weakly positive** | TAF | august | no |  |
| RO-Tac-BR | BR | *Culicoides punctatus* | 10 | 0 |  | TAF | august | no |  |
| RO-Tac-BR | BR | *Culicoides punctatus* | 10 | 0 |  | TAF | august | no |  |
| RO-Tac-BR | BR | *Culicoides punctatus* | 10 | 0 |  | TAF | august | no |  |
| RO-Domn-AG | AG | *Stomoxys calcitrans* | 2 | 32.9 | **weakly positive** | CF | august | yes | *Sus scrofa* |
| RO-Domn-AG | AG | *Stomoxys calcitrans* | 2 | 0 |  | CF | august | yes |  |
| RO-Domn-AG | AG | *Stomoxys calcitrans* | 2 | 28.87 | **positive** | CF | august | yes |  |
| RO-Domn-AG | AG | *Culicoides obsoletus* | 10 | 0 |  | CF | august | yes |  |
| RO-Domn-AG | AG | *Culicoides newsteadi* | 10 | 0 |  | CF | august | yes |  |
| RO-Domn-AG | AG | *Culicoides pulicaris* | 10 | 0 |  | CF | august | yes |  |
| RO-Domn-AG | AG | *Culicoides pulicaris* | 10 | 0 |  | CF | august | yes |  |
| RO-Domn-AG | AG | *Culicoides pulicaris* | 10 | 38.24 | **weakly positive** | CF | august | yes | *Sus scrofa* |
| RO-Domn-AG | AG | *Culicoides punctatus* | 10 | 27.93 | **positive** | CF | august | yes | *Sus scrofa* |
| RO-Domn-AG | AG | *Culicoides punctatus* | 10 | 30.28 | **weakly positive** | CF | august | yes | *Sus scrofa* |
| RO-Domn-AG | AG | *Culicoides punctatus* | 10 | 0 |  | CF | august | yes |  |
| RO-Domn-AG | AG | *Culicoides punctatus* | 10 | 0 |  | CF | august | yes |  |
| RO-Domn-AG | AG | *Culicoides obsoletus* | 10 | 28.95 | **positive** | CF | august | yes | *Sus scrofa* |
| RO-Domn-AG | AG | *Culicoides obsoletus* | 10 | 0 |  | CF | august | yes |  |
| RO-Domn-AG | AG | *Culicoides obsoletus* | 10 | 30.14 | **weakly positive** | CF | august | yes | *Sus scrofa* |
| RO-Domn-AG | AG | *Culicoides obsoletus* | 10 | 30.35 | **weakly positive** | CF | august | yes | *Sus scrofa* |
| RO-Domn-AG | AG | *Culicoides obsoletus* | 10 | 32.66 | **weakly positive** | CF | august | yes | *Sus scrofa* |
| RO-Domn-AG | AG | *Culicoides obsoletus* | 10 | 31.96 | **weakly positive** | CF | august | yes | *Sus scrofa* |
| RO-Domn-AG | AG | *Culicoides obsoletus* | 10 | 0 |  | CF | august | yes |  |
| RO-Domn-AG | AG | *Culicoides obsoletus* | 10 | 32.94 | **weakly positive** | CF | august | yes | *Sus scrofa* |
| RO-Domn-AG | AG | *Culicoides obsoletus* | 10 | 31.12 | **weakly positive** | CF | august | yes | *Sus scrofa* |
| RO-Domn-AG | AG | *Culicoides obsoletus* | 10 | 29.21 | **positive** | CF | august | yes | *Sus scrofa* |
| RO-Domn-AG | AG | *Culicoides obsoletus* | 10 | 32.37 | **weakly positive** | CF | august | yes | *Sus scrofa* |
| RO-Domn-AG | AG | *Culicoides obsoletus* | 10 | 38.82 | **weakly positive** | CF | august | yes | *Sus scrofa* |
| RO-Domn-AG | AG | *Culicoides obsoletus* | 10 | 34.75 | **weakly positive** | CF | august | yes | *Sus scrofa* |
| RO-Domn-AG | AG | *Culicoides obsoletus* | 10 | 31.79 | **weakly positive** | CF | august | yes | *Sus scrofa* |
| RO-Domn-AG | AG | *Culicoides obsoletus* | 10 | 0 |  | CF | august | yes |  |
| RO-Domn-AG | AG | *Culicoides obsoletus* | 10 | 0 |  | CF | august | yes |  |
| RO-Domn-AG | AG | *Culicoides obsoletus* | 10 | 0 |  | CF | august | yes |  |
| RO-Domn-AG | AG | *Culicoides obsoletus* | 10 | 38.41 | **weakly positive** | CF | august | yes | *Sus scrofa* |
| RO-Domn-AG | AG | *Culicoides obsoletus* | 10 | 31.32 | **weakly positive** | CF | august | yes | *Sus scrofa* |
| RO-Domn-AG | AG | *Culicoides obsoletus* | 10 | 32.73 | **weakly positive** | CF | august | yes | *Sus scrofa* |
| RO-Domn-AG | AG | *Culicoides obsoletus* | 10 | 31.52 | **weakly positive** | CF | august | yes | *Sus scrofa* |
| RO-Domn-AG | AG | *Culicoides obsoletus* | 10 | 0 |  | CF | august | yes |  |
| RO-Domn-AG | AG | *Culicoides obsoletus* | 10 | 0 |  | CF | august | yes |  |
| RO-Domn-AG | AG | *Culicoides obsoletus* | 10 | 27.72 | **positive** | CF | august | yes | *Sus scrofa* |
| RO-Domn-AG | AG | *Culicoides obsoletus* | 10 | 33.31 | **weakly positive** | CF | august | yes | *Sus scrofa* |
| RO-Domn-AG | AG | *Culicoides obsoletus* | 10 | 27.16 | **positive** | CF | august | yes | *Sus scrofa* |
| RO-Domn-AG | AG | *Culicoides obsoletus* | 10 | 31.46 | **weakly positive** | CF | august | yes |  |
| RO-Domn-AG | AG | *Culicoides obsoletus* | 10 | 30.66 | **weakly positive** | CF | august | yes | *Sus scrofa* |
| RO-Domn-AG | AG | *Culicoides obsoletus* | 10 | 0 |  | CF | august | yes |  |
| RO-Domn-AG | AG | *Culicoides obsoletus* | 10 | 32.61 | **weakly positive** | CF | august | yes | *Sus scrofa* |
| RO-Domn-AG | AG | *Culicoides obsoletus* | 10 | 31.49 | **weakly positive** | CF | august | yes |  |
| RO-Domn-AG | AG | *Culicoides obsoletus* | 10 | 30.54 | **weakly positive** | CF | august | yes |  |
| RO-Domn-AG | AG | *Culicoides obsoletus* | 10 | 35.04 | **weakly positive** | CF | august | yes | *Sus scrofa* |
| RO-Cer-TR | TR | *Culicoides nubeculosus* | 10 | 0 |  | BF | september | yes |  |
| RO-Cer-TR | TR | *Culicoides nubeculosus* | 10 | 0 |  | BF | september | yes |  |
| RO-Cer-TR | TR | *Culicoides nubeculosus* | 10 | 0 |  | BF | september | yes |  |
| RO-Cer-TR | TR | *Culicoides nubeculosus* | 10 | 0 |  | BF | september | yes |  |
| RO-Cer-TR | TR | *Culicoides nubeculosus* | 10 | 0 |  | BF | september | yes |  |
| RO-Cer-TR | TR | *Culicoides nubeculosus* | 10 | 0 |  | BF | september | yes |  |
| RO-Cer-TR | TR | *Culicoides nubeculosus* | 10 | 0 |  | BF | september | yes |  |
| RO-Cer-TR | TR | *Culicoides nubeculosus* | 10 | 0 |  | BF | september | yes |  |
| RO-Cer-TR | TR | *Culicoides nubeculosus* | 10 | 0 |  | BF | september | yes |  |
| RO-Cer-TR | TR | *Culicoides newsteadi* | 10 | 0 |  | BF | september | yes |  |
| RO-Cer-TR | TR | *Culicoides newsteadi* | 10 | 0 |  | BF | september | yes |  |
| RO-Cer-TR | TR | *Culicoides newsteadi* | 10 | 0 |  | BF | september | yes |  |
| RO-Cer-TR | TR | *Culicoides newsteadi* | 10 | 0 |  | BF | september | yes |  |
| RO-Cer-TR | TR | *Culicoides obsoletus* | 10 | 0 |  | BF | september | yes |  |
| RO-Cer-TR | TR | *Culicoides circumscriptus* | 10 | 0 |  | BF | september | yes |  |
| RO-Cer-TR | TR | *Culicoides festivipennis* | 10 | 0 |  | BF | september | yes |  |
| RO-Fra-OT | OT | *Culicoides circumscriptus* | 10 | 0 |  | BF | september | no |  |
| RO-Fra-OT | OT | *Culicoides festivipennis* | 10 | 0 |  | BF | september | no |  |
| RO-Fra-OT | OT | *Culicoides obsoletus* | 10 | 0 |  | BF | september | no |  |
| RO-Fra-OT | OT | *Culicoides newsteadi* | 10 | 0 |  | BF | september | no |  |
| RO-Fra-OT | OT | *Culicoides newsteadi* | 10 | 0 |  | BF | september | no |  |
| RO-Fra-OT | OT | *Culicoides newsteadi* | 10 | 0 |  | BF | september | no |  |
| RO-Fra-OT | OT | *Culicoides nubeculosus* | 10 | 0 |  | BF | september | no |  |
| RO-Fra-OT | OT | *Culicoides punctatus* | 10 | 0 |  | BF | september | no |  |
| RO-Poc-GJ | GJ | *Culicoides festivipennis* | 10 | 0 |  | BF | september | no |  |
| RO-Poc-GJ | GJ | *Culicoides punctatus* | 10 | 0 |  | BF | september | no |  |
| RO-Poc-GJ | GJ | *Culicoides obsoletus* | 10 | 0 |  | BF | september | no |  |
| RO-Poc-GJ | GJ | *Culicoides obsoletus* | 10 | 0 |  | BF | september | no |  |
| RO-Poc-GJ | GJ | *Culicoides nubeculosus* | 10 | 0 |  | BF | september | no |  |
| RO-Poc-GJ | GJ | *Culicoides nubeculosus* | 10 | 0 |  | BF | september | no |  |
| RO-Poc-GJ | GJ | *Culicoides newsteadi* | 10 | 0 |  | BF | september | no |  |
| RO-Poc-GJ | GJ | *Culicoides newsteadi* | 10 | 0 |  | BF | september | no |  |
| RO-Poc-GJ | GJ | *Culicoides circumscriptus* | 10 | 0 |  | BF | september | no |  |
| RO-Poc-GJ | GJ | *Culicoides circumscriptus* | 10 | 0 |  | BF | september | no |  |
| RO-Poc-GJ | GJ | *Culicoides circumscriptus* | 10 | 0 |  | BF | september | no |  |
| RO-Poc-GJ | GJ | *Culicoides circumscriptus* | 10 | 0 |  | BF | september | no |  |
| RO-Poc-GJ | GJ | *Culicoides circumscriptus* | 10 | 0 |  | BF | september | no |  |
| RO-Poc-GJ | GJ | *Culicoides circumscriptus* | 10 | 0 |  | BF | september | no |  |
| RO-Poc-GJ | GJ | *Culicoides obsoletus* | 10 | 0 |  | BF | september | no |  |
| RO-Poc-GJ | GJ | *Culicoides obsoletus* | 10 | 0 |  | BF | september | no |  |
| RO-Poc-GJ | GJ | *Culicoides obsoletus* | 10 | 0 |  | BF | september | no |  |
| RO-Poc-GJ | GJ | *Culicoides obsoletus* | 10 | 0 |  | BF | september | no |  |
| RO-Poc-GJ | GJ | *Culicoides obsoletus* | 10 | 0 |  | BF | september | no |  |
| RO-Poc-GJ | GJ | *Culicoides obsoletus* | 10 | 0 |  | BF | september | no |  |
| RO-Poc-GJ | GJ | *Culicoides obsoletus* | 10 | 0 |  | BF | september | no |  |
| RO-Poc-GJ | GJ | *Culicoides obsoletus* | 10 | 0 |  | BF | september | no |  |
| RO-Poc-GJ | GJ | *Culicoides obsoletus* | 10 | 0 |  | BF | september | no |  |
| RO-Poc-GJ | GJ | *Culicoides obsoletus* | 10 | 0 |  | BF | september | no |  |
| RO-Poc-GJ | GJ | *Culicoides obsoletus* | 10 | 0 |  | BF | september | no |  |
| RO-Poc-GJ | GJ | *Culicoides obsoletus* | 10 | 0 |  | BF | september | no |  |
| RO-Poc-GJ | GJ | *Culicoides obsoletus* | 10 | 0 |  | BF | september | no |  |
| RO-Poc-GJ | GJ | *Culicoides obsoletus* | 10 | 0 |  | BF | september | no |  |
| RO-Poc-GJ | GJ | *Culicoides obsoletus* | 10 | 0 |  | BF | september | no |  |
| RO-Poc-GJ | GJ | *Culicoides obsoletus* | 10 | 0 |  | BF | september | no |  |
| RO-Poc-GJ | GJ | *Culicoides obsoletus* | 10 | 0 |  | BF | september | no |  |
| RO-Poc-GJ | GJ | *Culicoides obsoletus* | 10 | 0 |  | BF | september | no |  |
| RO-Poc-GJ | GJ | *Culicoides obsoletus* | 10 | 0 |  | BF | september | no |  |
| RO-Poc-GJ | GJ | *Culicoides obsoletus* | 10 | 0 |  | BF | september | no |  |
| RO-Poc-GJ | GJ | *Culicoides obsoletus* | 10 | 0 |  | BF | september | no |  |
| RO-Poc-GJ | GJ | *Culicoides obsoletus* | 10 | 0 |  | BF | september | no |  |
| RO-Poc-GJ | GJ | *Culicoides obsoletus* | 10 | 0 |  | BF | september | no |  |
| RO-Poc-GJ | GJ | *Culicoides obsoletus* | 10 | 0 |  | BF | september | no |  |
| RO-Poc-GJ | GJ | *Culicoides obsoletus* | 10 | 0 |  | BF | september | no |  |
| RO-Poc-GJ | GJ | *Culicoides obsoletus* | 10 | 0 |  | BF | september | no |  |
| RO-Poc-GJ | GJ | *Culicoides obsoletus* | 10 | 0 |  | BF | september | no |  |
| RO-Poc-GJ | GJ | *Culicoides obsoletus* | 10 | 0 |  | BF | september | no |  |
| RO-Poc-GJ | GJ | *Culicoides lupicaris* | 10 | 0 |  | BF | september | no |  |
| RO-Poc-GJ | GJ | *Culicoides lupicaris* | 10 | 0 |  | BF | september | no |  |
| RO-Poc-GJ | GJ | *Culicoides lupicaris* | 10 | 0 |  | BF | september | no |  |
| RO-Poc-GJ | GJ | *Culicoides lupicaris* | 10 | 0 |  | BF | september | no |  |
| RO-Poc-GJ | GJ | *Culicoides lupicaris* | 10 | 0 |  | BF | september | no |  |
| RO-Poc-GJ | GJ | *Culicoides lupicaris* | 10 | 0 |  | BF | september | no |  |
| RO-Poc-GJ | GJ | *Culicoides lupicaris* | 10 | 0 |  | BF | september | no |  |
| RO-Poc-GJ | GJ | *Culicoides lupicaris* | 10 | 0 |  | BF | september | no |  |
| RO-Poc-GJ | GJ | *Culicoides lupicaris* | 10 | 0 |  | BF | september | no |  |
| RO-Poc-GJ | GJ | *Culicoides lupicaris* | 10 | 0 |  | BF | september | no |  |
| RO-Poc-GJ | GJ | *Culicoides lupicaris* | 10 | 0 |  | BF | september | no |  |
| RO-Poc-GJ | GJ | *Culicoides lupicaris* | 10 | 0 |  | BF | september | no |  |
| RO-Poc-GJ | GJ | *Culicoides lupicaris* | 10 | 0 |  | BF | september | no |  |
| RO-Poc-GJ | GJ | *Culicoides lupicaris* | 10 | 0 |  | BF | september | no |  |
| RO-Poc-GJ | GJ | *Culicoides lupicaris* | 10 | 0 |  | BF | september | no |  |
| RO-Poc-GJ | GJ | *Culicoides lupicaris* | 10 | 0 |  | BF | september | no |  |
| RO-Poc-GJ | GJ | *Culicoides lupicaris* | 10 | 0 |  | BF | september | no |  |
| RO-Poc-GJ | GJ | *Culicoides lupicaris* | 10 | 0 |  | BF | september | no |  |
| RO-Poc-GJ | GJ | *Culicoides lupicaris* | 10 | 0 |  | BF | september | no |  |
| RO-Poc-GJ | GJ | *Culicoides lupicaris* | 10 | 0 |  | BF | september | no |  |
| RO-Poc-GJ | GJ | *Culicoides lupicaris* | 10 | 0 |  | BF | september | no |  |
| RO-Poc-GJ | GJ | *Culicoides lupicaris* | 10 | 0 |  | BF | september | no |  |
| RO-Poc-GJ | GJ | *Culicoides lupicaris* | 10 | 0 |  | BF | september | no |  |
| RO-Poc-GJ | GJ | *Culicoides lupicaris* | 10 | 0 |  | BF | september | no |  |
| RO-Poc-GJ | GJ | *Culicoides lupicaris* | 10 | 0 |  | BF | september | no |  |
| RO-Poc-GJ | GJ | *Culicoides lupicaris* | 10 | 0 |  | BF | september | no |  |
| RO-Poc-GJ | GJ | *Culicoides lupicaris* | 10 | 0 |  | BF | september | no |  |
| RO-Sir-AR | AR | *Stomoxys calcitrans* | 2 | 0 |  | TAF | august | no |  |
| RO-Sir-AR | AR | *Stomoxys calcitrans* | 2 | 26.13 | **positive** | TAF | august | no |  |
| RO-Sir-AR | AR | *Stomoxys calcitrans* | 2 | 30.84 | **weakly positive** | TAF | august | no | *Sus scrofa* |
| RO-Sir-AR | AR | *Stomoxys calcitrans* | 2 | 0 |  | TAF | august | no |  |
| RO-Sir-AR | AR | *Culicoides obsoletus* | 10 | 0 |  | TAF | august | no |  |
| RO-Sir-AR | AR | *Culicoides newsteadi* | 10 | 0 |  | TAF | august | no |  |
| RO-Sir-AR | AR | *Culicoides nubeculosus* | 10 | 0 |  | TAF | august | no |  |
| RO-Sir-AR | AR | *Culicoides nubeculosus* | 10 | 0 |  | TAF | august | no |  |
| RO-Sir-AR | AR | *Culicoides nubeculosus* | 10 | 0 |  | TAF | august | no |  |
| RO-Cal-CL | CL | *Stomoxys calcitrans* | 2 | 26.55 | **positive** | CF | august | yes | *Homo sapiens* |
| RO-Cal-CL | CL | *Stomoxys calcitrans* | 2 | 32.23 | **weakly positive** | CF | august | yes | *Homo sapiens* |
| RO-Cal-CL | CL | *Stomoxys calcitrans* | 2 | 0 |  | CF | august | yes |  |
| RO-Cal-CL | CL | *Stomoxys calcitrans* | 2 | 37.3 | **weakly positive** | CF | august | yes |  |
| RO-Cal-CL | CL | *Culicoides punctatus* | 10 | 0 |  | CF | august | yes |  |
| RO-Cur-AR | AR | *Culicoides nubeculosus* | 10 | 0 |  | BF | august | no |  |
| RO-Cur-AR | AR | *Culicoides nubeculosus* | 10 | 0 |  | BF | august | no |  |
| RO-Cur-AR | AR | *Culicoides obsoletus* | 10 | 0 |  | BF | august | no |  |
| RO-Cur-AR | AR | *Stomoxys calcitrans* | 2 | 0 |  | BF | august | no |  |
| RO-Tac-BR | BR | *Culicoides obsoletus* | 10 | 0 |  | TAF | august | no |  |
| RO-Pau-VN | VN | *Culicoides obsoletus* | 10 | 0 |  | TAF | july | no |  |
| RO-SEI-MM | MM | *Culicoides obsoletus* | 10 | 29.78 | **positive** | TAF | august | no |  |
| RO-SEI-MM | MM | *Stomoxys calcitrans* | 2 | 0 |  | TAF | august | no |  |
| RO-SEI-MM | MM | *Stomoxys calcitrans* | 2 | 0 |  | TAF | august | no |  |
| RO-SEI-MM | MM | *Stomoxys calcitrans* | 2 | 0 |  | TAF | august | no |  |
| RO-Dan -Ch-MM | MM | *Culicoides obsoletus* | 10 | 0 |  | BF | august | no |  |
| RO-Dan -Ch-MM | MM | *Culicoides obsoletus* | 10 | 0 |  | BF | august | no |  |
| RO-Dan -Ch-MM | MM | *Culicoides obsoletus* | 10 | 0 |  | BF | august | no |  |
| RO-Dan -Ch-MM | MM | *Culicoides obsoletus* | 10 | 0 |  | BF | august | no |  |
| RO-Tac-BR | BR | *Culicoides newsteadi* | 10 | 0 |  | TAF | august | no |  |
| RO-Domn-AG | AG | *Culicoides obsoletus* | 10 | 0 |  | CF | august | yes |  |
| RO-BR- BR | BR | *Culicoides obsoletus* | 10 | 0 |  | BF | august | no |  |
| RO-BR- BR | BR | *Culicoides obsoletus* | 10 | 35.72 | **weakly positive** | BF | august | no |  |
| RO-BR- BR | BR | *Culicoides obsoletus* | 10 | 0 |  | BF | august | no |  |
| RO-BR- BR | BR | *Culicoides obsoletus* | 10 | 0 |  | BF | august | no |  |
| RO-Bud BH | BH | *Culicoides punctatus* | 10 | 0 |  | BF | june | no |  |
| RO-Bud BH | BH | *Culicoides nubeculosus* | 10 | 0 |  | BF | june | no |  |
| RO-Pir-SM | SM | *Culicoides obsoletus* | 10 | 0 |  | BF | june | no |  |
| RO-Mal-VL | VL | *Culicoides circumscriptus* | 10 | 0 |  | BF | july | no |  |
| RO-Mal-VL | VL | *Culicoides submaritimus* | 10 | 0 |  | BF | july | no |  |
| RO-Cod-BV | BV | *Culicoides obsoletus* | 10 | 0 |  | CF | june | no |  |
| RO-Fra-OT | OT | *Culicoides newsteadi* | 10 | 0 |  | BF | september | no |  |
| RO-Rec-TM | TM | *Stomoxys calcitrans* | 2 | 22.68 | **strongly positive** | TAF | june | yes |  |
| RO-Rec-TM | TM | *Stomoxys calcitrans* | 2 | 23.87 | **strongly positive** | TAF | june | yes |  |
| RO-Rec-TM | TM | *Stomoxys calcitrans* | 2 | 22.74 | **strongly positive** | TAF | june | yes |  |
| RO-Rec-TM | TM | *Stomoxys calcitrans* | 2 | 20.51 | **strongly positive** | TAF | june | yes |  |
| RO-Rec-TM | TM | *Culicoides punctatus* | 10 | 0 |  | TAF | june | yes |  |
| RO-Rec-TM | TM | *Culicoides obsoletus* | 10 | 0 |  | TAF | june | yes |  |
| RO-Rec-TM | TM | *Culicoides nubeculosus* | 10 | 0 |  | TAF | june | yes |  |
| RO-Rec-TM | TM | *Culicoides newsteadi* | 10 | 0 |  | TAF | june | yes |  |
| RO-Cal-CL | CL | *Stomoxys calcitrans* | 2 | 21.36 | **strongly positive** | CF | august | yes |  |
| RO-Cal-CL | CL | *Stomoxys calcitrans* | 2 | 21.4 | **strongly positive** | CF | august | yes |  |
| RO-Cal-CL | CL | *Stomoxys calcitrans* | 2 | 24.73 | **positive** | CF | august | yes |  |
| RO-Cal-CL | CL | *Stomoxys calcitrans* | 2 | 19.84 | **strongly positive** | CF | august | yes |  |
| RO-Cal-CL | CL | *Stomoxys calcitrans* | 2 | 19.25 | **strongly positive** | CF | august | yes | *Sus scrofa* |
| RO-Cal-CL | CL | *Stomoxys calcitrans* | 2 | 27.59 | **positive** | CF | august | yes |  |
| RO-Cal-CL | CL | *Stomoxys calcitrans* | 2 | 25.06 | **positive** | CF | august | yes |  |
| RO-Cal-CL | CL | *Stomoxys calcitrans* | 2 | 29.95 | **positive** | CF | august | yes |  |
| RO-Cal-CL | CL | *Stomoxys calcitrans* | 2 | 23.31 | **strongly positive** | CF | august | yes |  |
| RO-Cal-CL | CL | *Stomoxys calcitrans* | 2 | 21.64 | **strongly positive** | CF | august | yes |  |
| RO-Cal-CL | CL | *Stomoxys calcitrans* | 2 | 18.19 | **strongly positive** | CF | august | yes |  |
| RO-Cal-CL | CL | *Stomoxys calcitrans* | 2 | 19.84 | **strongly positive** | CF | august | yes |  |
| RO-Cal-CL | CL | *Stomoxys calcitrans* | 2 | 20.37 | **strongly positive** | CF | august | yes |  |
| RO-Cal-CL | CL | *Stomoxys calcitrans* | 2 | 23.36 | **strongly positive** | CF | august | yes |  |
| RO-Cal-CL | CL | *Stomoxys calcitrans* | 2 | 20.17 | **strongly positive** | CF | august | yes |  |
| RO-Cal-CL | CL | *Stomoxys calcitrans* | 2 | 20.73 | **strongly positive** | CF | august | yes |  |
| RO-Cal-CL | CL | *Stomoxys calcitrans* | 2 | 20.87 | **strongly positive** | CF | august | yes |  |
| RO-Cal-CL | CL | *Stomoxys calcitrans* | 2 | 28.3 | **positive** | CF | august | yes | *Sus scrofa* |
| RO-Cal-CL | CL | *Stomoxys calcitrans* | 2 | 22.33 | **strongly positive** | CF | august | yes | *Lepus capensis* |
| RO-Cal-CL | CL | *Stomoxys calcitrans* | 2 | 21.61 | **strongly positive** | CF | august | yes | *Sus scrofa* |
| RO-Cal-CL | CL | *Stomoxys calcitrans* | 2 | 19.11 | **strongly positive** | CF | august | yes | *Sus scrofa* |
| RO-Cal-CL | CL | *Stomoxys calcitrans* | 2 | 21.85 | **strongly positive** | CF | august | yes |  |
| RO-Cal-CL | CL | *Culicoides obsoletus* | 10 | 0 |  | CF | august | yes |  |
| RO-Cal-CL | CL | *Culicoides punctatus* | 10 | 0 |  | CF | august | yes |  |
| RO-Cal-CL | CL | *Culicoides pulicaris* | 10 | 0 |  | CF | august | yes |  |
| RO-Cal-CL | CL | *Culicoides newsteadi* | 10 | 0 |  | CF | august | yes |  |
| RO-Fun-BZ | BZ | *Culicoides punctatus* | 10 | 0 |  | CF | september | yes |  |
| RO-Fun-BZ | BZ | *Culicoides punctatus* | 10 | 33.19 | **weakly positive** | CF | september | yes | *Mus musculus* |
| RO-Fun-BZ | BZ | *Culicoides punctatus* | 10 | 28.53 | **positive** | CF | september | yes | *Mus musculus* |
| RO-Fun-BZ | BZ | *Culicoides punctatus* | 10 | 31.42 | **weakly positive** | CF | september | yes |  |
| RO-Fun-BZ | BZ | *Culicoides punctatus* | 10 | 0 |  | CF | september | yes |  |
| RO-Fun-BZ | BZ | *Culicoides punctatus* | 10 | 0 |  | CF | september | yes |  |
| RO-Fun-BZ | BZ | *Culicoides punctatus* | 10 | 34.58 | **weakly positive** | CF | september | yes |  |
| RO-Fun-BZ | BZ | *Culicoides punctatus* | 10 | 0 |  | CF | september | yes |  |
| RO-Fun-BZ | BZ | *Culicoides punctatus* | 10 | 27.44 | **positive** | CF | september | yes |  |
| RO-Fun-BZ | BZ | *Culicoides punctatus* | 10 | 35.13 | **weakly positive** | CF | september | yes | *Mus musculus* |
| RO-Fun-BZ | BZ | *Culicoides punctatus* | 10 | 33.95 | **weakly positive** | CF | september | yes | *Mus musculus* |
| RO-Fun-BZ | BZ | *Culicoides punctatus* | 10 | 0 |  | CF | september | yes |  |
| RO-Fun-BZ | BZ | *Culicoides punctatus* | 10 | 0 |  | CF | september | yes |  |
| RO-Fun-BZ | BZ | *Culicoides punctatus* | 10 | 31.76 | **weakly positive** | CF | september | yes |  |
| RO-Fun-BZ | BZ | *Culicoides punctatus* | 10 | 0 |  | CF | september | yes |  |
| RO-Fun-BZ | BZ | *Culicoides punctatus* | 10 | 29.65 | **positive** | CF | september | yes |  |
| RO-Fun-BZ | BZ | *Culicoides punctatus* | 10 | 27.03 | **positive** | CF | september | yes |  |
| RO-Fun-BZ | BZ | *Culicoides punctatus* | 10 | 27.53 | **positive** | CF | september | yes |  |
| RO-Fun-BZ | BZ | *Culicoides punctatus* | 10 | 27.35 | **positive** | CF | september | yes |  |
| RO-Fun-BZ | BZ | *Culicoides punctatus* | 10 | 0 |  | CF | september | yes |  |
| RO-Fun-BZ | BZ | *Culicoides punctatus* | 10 | 30.26 | **weakly positive** | CF | september | yes | *Mus musculus* |
| RO-Fun-BZ | BZ | *Culicoides nubeculosus* | 10 | 0 |  | CF | september | yes |  |
| RO-Fun-BZ | BZ | *Culicoides festivipennis* | 10 | 0 |  | CF | september | yes |  |
| RO-Fun-BZ | BZ | *Culicoides newsteadi* | 10 | 0 |  | CF | september | yes |  |
| RO-Rec-TM | TM | *Culicoides circumscriptus* | 10 | 0 |  | TAF | june | yes |  |
| RO-Seac.C-DJ | DJ | *Culicoides obsoletus* | 10 | 0 |  | BF | july | no |  |
| RO-Ciup.N-DJ | DJ | *Stomoxys calcitrans* | 2 | 21.41 | **strongly positive** | BF | july | yes |  |
| RO-Ciup.N-DJ | DJ | *Stomoxys calcitrans* | 2 | 21.91 | **strongly positive** | BF | july | yes |  |
| RO-Ciup.N-DJ | DJ | *Stomoxys calcitrans* | 2 | 22.37 | **strongly positive** | BF | july | yes | *Mus musculus* |
| RO-Ciup.N-DJ | DJ | *Stomoxys calcitrans* | 2 | 20.5 | **strongly positive** | BF | july | yes | *Mus musculus* |
| RO-Ciup.N-DJ | DJ | *Stomoxys calcitrans* | 2 | 39.53 | **weakly positive** | BF | july | yes | *Bos taurus* |
| RO-Sir-Ar | AR | *Culicoides newsteadi* | 10 | 0 |  | TAF | august | no |  |
| RO-Sir-Ar | AR | *Culicoides nubeculosus* | 10 | 33.98 | **weakly positive** | TAF | august | no |  |
| RO-Sir-Ar | AR | *Culicoides nubeculosus* | 10 | 0 |  | TAF | august | no |  |
| RO-Sir-Ar | AR | *Culicoides obsoletus* | 10 | 0 |  | TAF | august | no |  |
| RO-Sir-Ar | AR | *Culicoides punctatus* | 10 | 0 |  | TAF | august | no |  |
| RO-Sir-Ar | AR | *Stomoxys calcitrans* | 2 | 24.64 | **positive** | TAF | august | no |  |
| RO-Sir-Ar | AR | *Stomoxys calcitrans* | 2 | 30.27 | **weakly positive** | TAF | august | no |  |
| RO-Cal-CL | CL | *Culicoides circumscriptus* | 10 | 0 |  | CF | august | yes |  |
| RO-Cal-CL | CL | *Culicoides nubeculosus* | 10 | 0 |  | CF | august | yes |  |
| RO-Cal-CL | CL | *Culicoides festivipennis* | 10 | 35.5 | **weakly positive** | CF | august | yes |  |
| RO-Cal-CL | CL | *Culicoides punctatus* | 10 | 0 |  | CF | august | yes |  |
| RO-Cal-CL | CL | *Stomoxys calcitrans* | 2 | 22.65 | **strongly positive** | CF | august | yes |  |
| RO-Bai-DJ | DJ | *Culicoides newsteadi* | 10 | 39.27 | **weakly positive** | CF | august | yes |  |
| RO-Bai-DJ | DJ | *Culicoides nubeculosus* | 10 | 0 |  | CF | august | yes |  |
| RO-Bai-DJ | DJ | *Stomoxys calcitrans* | 2 | 35.64 | **weakly positive** | CF | august | yes |  |
| RO-Fra-OT | OT | *Culicoides festivipennis* | 10 | 0 |  | BF | september | no |  |
| RO-Fra-OT | OT | *Culicoides newsteadi* | 10 | 0 |  | BF | september | no |  |
| RO-Fra-OT | OT | *Culicoides newsteadi* | 10 | 34.42 | **weakly positive** | BF | september | no |  |
| RO-Fra-OT | OT | *Culicoides punctatus* | 10 | 0 |  | BF | september | no |  |
| RO-Fra-OT | OT | *Culicoides obsoletus* | 10 | 0 |  | BF | september | no |  |
| RO-Fra-OT | OT | *Culicoides puncticollis* | 10 | 36.59 | **weakly positive** | BF | september | no |  |
| RO-Fra-OT | OT | *Culicoides obsoletus* | 10 | 0 |  | BF | september | no |  |
| RO-Fra-OT | OT | *Culicoides pulicaris* | 10 | 0 |  | BF | september | no |  |
| RO-Fra-OT | OT | *Culicoides nubeculosus* | 10 | 34.88 | **weakly positive** | BF | september | no |  |
| RO-Fra-OT | OT | *Stomoxys calcitrans* | 2 | 0 |  | BF | september | no |  |
| RO-Fra-OT | OT | *Stomoxys calcitrans* | 2 | 0 |  | BF | september | no |  |
| RO-Fra-OT | OT | *Stomoxys calcitrans* | 2 | 35.21 | **weakly positive** | BF | september | no | *Mus musculus* |
| RO-Poc-GJ | GJ | *Stomoxys calcitrans* | 2 | 0 |  | BF | september | no |  |
| RO-Poc-GJ | GJ | *Culicoides submaritimus* | 10 | 34.52 | **weakly positive** | BF | september | no |  |
| RO-Poc-GJ | GJ | *Culicoides obsoletus* | 10 | 0 |  | BF | september | no |  |
| RO-Poc-GJ | GJ | *Culicoides lupicaris* | 10 | 34.4 | **weakly positive** | BF | september | no |  |
| RO-Poc-GJ | GJ | *Culicoides lupicaris* | 10 | 0 |  | BF | september | no |  |
| RO-Poc-GJ | GJ | *Culicoides obsoletus* | 10 | 0 |  | BF | september | no |  |
| RO-Poc-GJ | GJ | *Culicoides obsoletus* | 10 | 0 |  | BF | september | no |  |
| RO-Cer-TR | TR | *Culicoides nubeculosus* | 10 | 33.88 | **weakly positive** | BF | september | yes |  |
| RO-Cer-TR | TR | *Culicoides nubeculosus* | 10 | 0 |  | BF | september | yes |  |
| RO-Cer-TR | TR | *Culicoides nubeculosus* | 10 | 34.42 | **weakly positive** | BF | september | yes |  |
| RO-Cer-TR | TR | *Culicoides nubeculosus* | 10 | 32.47 | **weakly positive** | BF | september | yes |  |
| RO-Cer-TR | TR | *Culicoides newsteadi* | 10 | 0 |  | BF | september | yes |  |
| RO-Cer-TR | TR | *Culicoides festivipennis* | 10 | 0 |  | BF | september | yes |  |
| RO-Cur-AR | AR | *Stomoxys calcitrans* | 2 | 35.92 | **weakly positive** | BF | august | no |  |
| RO-Cur-AR | AR | *Stomoxys calcitrans* | 2 | 35.64 | **weakly positive** | BF | august | no |  |
| RO-Cur-AR | AR | *Stomoxys calcitrans* | 2 | 0 |  | BF | august | no |  |
| RO-Cur-AR | AR | *Stomoxys calcitrans* | 2 | 0 |  | BF | august | no |  |
| RO-Cur-AR | AR | *Stomoxys calcitrans* | 2 | 0 |  | BF | august | no |  |
| RO-Cur-AR | AR | *Stomoxys calcitrans* | 2 | 35.64 | **weakly positive** | BF | august | no |  |
| RO-Cur-AR | AR | *Stomoxys calcitrans* | 2 | 34.6 | **weakly positive** | BF | august | no |  |
| RO-Cur-AR | AR | *Stomoxys calcitrans* | 2 | 35.79 | **weakly positive** | BF | august | no |  |
| RO-Cur-AR | AR | *Stomoxys calcitrans* | 2 | 33.74 | **weakly positive** | BF | august | no |  |
| RO-Cur-AR | AR | *Culicoides nubeculosus* | 10 | 0 |  | BF | august | no |  |
| RO-Cur-AR | AR | *Culicoides nubeculosus* | 10 | 0 |  | BF | august | no |  |
| RO-Cur-AR | AR | *Culicoides nubeculosus* | 10 | 0 |  | BF | august | no |  |
| RO-Cur-AR | AR | *Culicoides nubeculosus* | 10 | 0 |  | BF | august | no |  |
| RO-Cur-AR | AR | *Culicoides nubeculosus* | 10 | 34.83 | **weakly positive** | BF | august | no |  |
| RO-Cur-AR | AR | *Culicoides nubeculosus* | 10 | 0 |  | BF | august | no |  |
| RO-Cur-AR | AR | *Culicoides nubeculosus* | 10 | 0 |  | BF | august | no |  |
| RO-Cur-AR | AR | *Culicoides obsoletus* | 10 | 35.16 | **weakly positive** | BF | august | no |  |
| RO-Cur-AR | AR | *Culicoides obsoletus* | 10 | 33.84 | **weakly positive** | BF | august | no | *Homo sapiens* |
| RO-Cur-AR | AR | *Culicoides obsoletus* | 10 | 0 |  | BF | august | no |  |
| RO-Sir-Ar | AR | *Stomoxys calcitrans* | 2 | 34.77 | **weakly positive** | TAF | august | no |  |
| RO-Sir-Ar | AR | *Stomoxys calcitrans* | 2 | 0 |  | TAF | august | no |  |
| RO-Sir-Ar | AR | *Stomoxys calcitrans* | 2 | 0 |  | TAF | august | no |  |
| RO-Sir-Ar | AR | *Stomoxys calcitrans* | 2 | 34.53 | **weakly positive** | TAF | august | no |  |
| RO-Sir-Ar | AR | *Culicoides nubeculosus* | 10 | 0 |  | TAF | august | no |  |
| RO-Sir-Ar | AR | *Culicoides nubeculosus* | 10 | 0 |  | TAF | august | no |  |
| RO-Sir-Ar | AR | *Culicoides nubeculosus* | 10 | 0 |  | TAF | august | no |  |
| RO-Sir-Ar | AR | *Culicoides newsteadi* | 10 | 34.6 | **weakly positive** | TAF | august | no |  |
| RO-Domn-AG | AG | *Culicoides obsoletus* | 10 | 30.15 | **weakly positive** | CF | august | yes |  |
| RO-Domn-AG | AG | *Culicoides obsoletus* | 10 | 32.24 | **weakly positive** | CF | august | yes |  |
| RO-Domn-AG | AG | *Culicoides obsoletus* | 10 | 0 |  | CF | august | yes |  |
| RO-Domn-AG | AG | *Culicoides obsoletus* | 10 | 29.43 | **positive** | CF | august | yes |  |
| RO-Domn-AG | AG | *Culicoides obsoletus* | 10 | 25.71 | **positive** | CF | august | yes |  |
| RO-Domn-AG | AG | *Culicoides obsoletus* | 10 | 28.7 | **positive** | CF | august | yes |  |
| RO-Domn-AG | AG | *Culicoides obsoletus* | 10 | 31.95 | **weakly positive** | CF | august | yes |  |
| RO-Domn-AG | AG | *Culicoides obsoletus* | 10 | 25.63 | **positive** | CF | august | yes |  |
| RO-Domn-AG | AG | *Culicoides obsoletus* | 10 | 29.96 | **positive** | CF | august | yes |  |
| RO-Domn-AG | AG | *Culicoides obsoletus* | 10 | 26.48 | **positive** | CF | august | yes |  |
| RO-Domn-AG | AG | *Culicoides obsoletus* | 10 | 31.41 | **weakly positive** | CF | august | yes | *Sus scrofa* |
| RO-Domn-AG | AG | *Culicoides obsoletus* | 10 | 33.74 | **weakly positive** | CF | august | yes |  |
| RO-Domn-AG | AG | *Culicoides obsoletus* | 10 | 0 |  | CF | august | yes |  |
| RO-Domn-AG | AG | *Culicoides obsoletus* | 10 | 30.9 | **weakly positive** | CF | august | yes |  |
| RO-Domn-AG | AG | *Culicoides obsoletus* | 10 | 27.31 | **positive** | CF | august | yes |  |
| RO-Domn-AG | AG | *Culicoides obsoletus* | 10 | 0 |  | CF | august | yes |  |
| RO-Domn-AG | AG | *Culicoides obsoletus* | 10 | 0 |  | CF | august | yes |  |
| RO-Domn-AG | AG | *Culicoides obsoletus* | 10 | 33.5 | **weakly positive** | CF | august | yes | *Sus scrofa* |
| RO-Domn-AG | AG | *Culicoides obsoletus* | 10 | 26.04 | **positive** | CF | august | yes | *Sus scrofa* |
| RO-Domn-AG | AG | *Culicoides obsoletus* | 10 | 34.77 | **weakly positive** | CF | august | yes | *Sus scrofa* |
| RO-Domn-AG | AG | *Culicoides obsoletus* | 10 | 27.37 | **positive** | CF | august | yes | *Sus scrofa* |
| RO-Domn-AG | AG | *Culicoides punctatus* | 10 | 34.18 | **weakly positive** | CF | august | yes | *Potamochoerus larvatus* |
| RO-Domn-AG | AG | *Culicoides punctatus* | 10 | 31.76 | **weakly positive** | CF | august | yes | *Sus scrofa* |
| RO-Domn-AG | AG | *Culicoides pulicaris* | 10 | 34.82 | **weakly positive** | CF | august | yes |  |
| RO-Domn-AG | AG | *Culicoides newsteadi* | 10 | 0 |  | CF | august | yes |  |
| RO-Domn-AG | AG | *Stomoxys calcitrans* | 2 | 0 |  | CF | august | yes |  |
| RO-Domn-AG | AG | *Stomoxys calcitrans* | 2 | 35.13 | **weakly positive** | CF | august | yes |  |
| RO-BR- BR | BR | *Culicoides obsoletus* | 10 | 0 |  | BF | august | no |  |
| RO-Tac-BR | BR | *Stomoxys calcitrans* | 2 | 0 |  | TAF | august | no |  |
| RO-Fun-BZ | BZ | *Stomoxys calcitrans* | 2 | 0 |  | CF | september | yes |  |
| RO-Fun-BZ | BZ | *Stomoxys calcitrans* | 2 | 35.7 | **weakly positive** | CF | september | yes |  |
| RO-Fun-BZ | BZ | *Stomoxys calcitrans* | 2 | 37.12 | **weakly positive** | CF | september | yes |  |
| RO-Gr-IL | IL | *Stomoxys calcitrans* | 2 | 0 |  | TAF | july | no |  |
| RO-Gr-IL | IL | *Stomoxys calcitrans* | 2 | 0 |  | TAF | july | no |  |
| RO-Gr-IL | IL | *Stomoxys calcitrans* | 2 | 0 |  | TAF | july | no |  |
| RO-Gr-IL | IL | *Stomoxys calcitrans* | 2 | 0 |  | TAF | july | no |  |
| RO-Gr-IL | IL | *Stomoxys calcitrans* | 2 | 0 |  | TAF | july | no |  |
| RO-Gr-IL | IL | *Stomoxys calcitrans* | 2 | 0 |  | TAF | july | no |  |
| RO-Gr-IL | IL | *Stomoxys calcitrans* | 2 | 0 |  | TAF | july | no |  |
| RO-Gr-IL | IL | *Stomoxys calcitrans* | 2 | 36.04 | **weakly positive** | TAF | july | no |  |
| RO-Gr-IL | IL | *Stomoxys calcitrans* | 2 | 0 |  | TAF | july | no |  |
| RO-Gr-IL | IL | *Stomoxys calcitrans* | 2 | 0 |  | TAF | july | no |  |
| RO-Dom-IF | IF | *Stomoxys calcitrans* | 2 | 0 |  | BF | june | no |  |
| RO-Dom-IF | IF | *Stomoxys calcitrans* | 2 | 0 |  | BF | june | no |  |
| RO-Dom-IF | IF | *Stomoxys calcitrans* | 2 | 0 |  | BF | june | no |  |
| RO-Dom-IF | IF | *Stomoxys calcitrans* | 2 | 0 |  | BF | june | no |  |
| RO-Dom-IF | IF | *Stomoxys calcitrans* | 2 | 0 |  | BF | june | no |  |
| RO-Dom-IF | IF | *Stomoxys calcitrans* | 2 | 37.57 | **weakly positive** | BF | june | no |  |
| RO-Dom-IF | IF | *Stomoxys calcitrans* | 2 | 0 |  | BF | june | no |  |
| RO-Dom-IF | IF | *Stomoxys calcitrans* | 2 | 0 |  | BF | june | no |  |
| RO-SEI-MM | MM | *Stomoxys calcitrans* | 2 | 35.92 | **weakly positive** | TAF | august | no |  |
| RO-Dan -Ch-MM | MM | *Culicoides obsoletus* | 10 | 0 |  | BF | august | no |  |
| RO-Rec-TM | TM | *Culicoides obsoletus* | 10 | 0 |  | TAF | june | yes |  |
| RO-Cer-TR | TR | *Culicoides obsoletus* | 10 | 0 |  | BF | september | yes |  |
| RO-Pau-VN | VN | *Stomoxys calcitrans* | 2 | 35.81 | **weakly positive** | TAF | july | no |  |
